# Costing the distribution of insecticide-treated nets: a review of cost and cost-effectiveness studies to provide guidance on standardization of costing methodology

**DOI:** 10.1186/1475-2875-5-37

**Published:** 2006-05-08

**Authors:** Jan Kolaczinski, Kara Hanson

**Affiliations:** 1 Disease Control and Vector Biology Unit, London School of Hygiene and Tropical Medicine, Keppel Street, London WC1E 7HT, UK; 2 Health Policy Unit, Department of Public Health and Policy, London School of Hygiene and Tropical Medicine, Keppel Street, London WC1E 7HT, UK

## Abstract

**Background:**

Insecticide-treated nets (ITNs) are an effective and cost-effective means of malaria control. Scaling-up coverage of ITNs is challenging. It requires substantial resources and there are a number of strategies to choose from. Information on the cost of different strategies is still scarce. To guide the choice of a delivery strategy (or combination of strategies), reliable and standardized cost information for the different options is required.

**Methods:**

The electronic online database PubMed was used for a systematic search of the published English literature on costing and economic evaluations of ITN distribution programmes. The keywords used were: net, bednet, insecticide, treated, ITN, cost, effectiveness, economic and evaluation. Identified papers were analysed to determine and evaluate the costing methods used. Methods were judged against existing standards of cost analysis to arrive at proposed standards for undertaking and presenting cost analyses.

**Results:**

Cost estimates were often not readily comparable or could not be adjusted to a different context. This resulted from the wide range of methods applied and measures of output chosen. Most common shortcomings were the omission of certain costs and failure to adjust financial costs to generate economic costs. Generalisability was hampered by authors not reporting quantities and prices of resources separately and not examining the sensitivity of their results to variations in underlying assumptions.

**Conclusion:**

The observed shortcomings have arisen despite the abundance of literature and guidelines on costing of health care interventions. This paper provides ITN specific recommendations in the hope that these will help to standardize future cost estimates.

## Introduction

Insecticide-treated nets (ITNs) are an effective tool for the prevention of morbidity and mortality caused by malaria [1] and other vector-borne diseases [2]. Cost-effectiveness of ITNs in the prevention of malaria has also been amply demonstrated in a variety of settings [3-5]. The present challenge is to scale-up and sustain coverage with ITNs [6]. Many approaches to ITN delivery have evolved. These vary widely in scale, target populations and in the strategy used to provide nets and insecticide to the end user. Recent advances in the development of nets with long-lasting insecticide impregnations (LLINs) simplify strategies by reducing the need for re-treatment [7,8].

To choose a delivery strategy (or combination of strategies), health planners and policy makers require reliable and standardised information on the cost per unit coverage [9]. Standardized costs are also needed for use in combination with effectiveness estimates to judge the efficiency of ITN programmes and the scope for their improvement. To generate data whereby alternative approaches can be compared a common set of techniques will need to be proposed and agreed upon [5,10]. The present study aims at contributing towards this process by reviewing the existing literature on costing and cost-effectiveness of ITN distribution programmes, identifying short-comings and suggesting ways to improve on quality and comparability of costing methods.

## Materials and methods

A systematic search of the published English literature on costing and economic evaluations of ITN distribution programmes was conducted by means of the electronic online database PubMed (US National Library of Medicine, Bethesda, USA). The keywords used were: net, bednet, insecticide, treated, ITN, cost, effectiveness, economic and evaluation. These searches were supplemented by iterative reviews of reference lists of relevant published papers. Two consultancy reports were also included.

All papers that provided cost estimates for the delivery of mosquito nets and/or insecticide treatment were considered relevant, even if they did not fully satisfy the criteria of a costing study [11]. To fulfil the aim of the present review of providing comprehensive guidance on how to improve and standardise costing of ITN distribution programmes, it was considered important to use cost and costing in their broader sense.

Studies were examined by means of a checklist adapted from other publications [12-14]. A summary of the following information was prepared: delivery and financing mechanism used, type of study (costing or cost-effectiveness), country where the study was implemented, perspective from which the authors measured costs, costs included, whether or not guidelines were used to assist in the identification, measurement and/or valuation of inputs, year of prices and results obtained. During this process, all studies that had been referred to by the authors as addressing cost-effectiveness were allocated to this category, rather than attempting to reclassify them to more precise categories such as those proposed elsewhere [11].

## Results

Twenty-six documents including two consultancy reports, published between 1989 and 2005, were identified as containing relevant cost estimates. Overall, 15 of the studies used an intermediate output (henceforth referred to as cost studies) whereas 11 related cost to a health outcome (cost-effectiveness studies) (Table 1).

**Table 1:** Summary of studies that provide cost estimates of the delivery of insecticide and/or nets

Category*	**Distribution**	Study**	**Country**	**Perspective**	**Cost included**		Guideline used***	**Year of price**	**Currency**	Cost****					**Source**
										
					**Financial**	**Economic**				Per net delivered	Per treatment delivered	Per ITN delivered	Per treated net year	Per person protected	
Public/Public	Campaign (Door-to-door)	C	Solomon Islands (Florida Islands)	Provider	Nets, insecticide, transport, fuel, repairs, wages, equipment, materials, facilities	Time taken	Yes	1988/89	SI$					3.85 (= US$ 1.97)	34^1^
	Campaign (Door-to-door)	C	China (Napo County)	Provider	Insecticide		No	1990 – 92	Yuan					0.48^F^	35
	Campaign (Door-to-door)	CE	Northern Ghana (Kassena – Nankana district)	Provider	Nets, insecticide, wages, transport, supplies & services	Adjusted financial costs plus time of volunteers	Yes	1993/94	US$ (and Cedis)			2.40			20
	General health facilities	CE	The Gambia	Provider	Nets, insecticide, etc. (taken from Picard *et al*. 1993)	Adjusted financial costs	No	1990	US$			6.24			36
	Campaign (Door-to-door)	C	Tanzania	Provider	Nets, insecticide, wages, transport	Cost of nets was annualised (but not discounted)	No	1996	US$	1.00^F^	0.46^F^	1.46^F^			37
	Campaign (Distrib. point not specified; assumed central)	CE	Thailand (Thai-Myanmar Border)	Patients	Direct medical costs, transportation, food	Time absent from work	Yes	1994/95	US$						38
	Campaign (Door-to-door)	C	Pakistan (Afghan refugee camps)	Provider	Net, insecticide, operational costs	Cost of net annualised (but not discounted)	No	1991 – 94	US$					1.51	39
	Campaign (Fixed-site)	CE	South Africa (KwaZulu-Natal)	Provider	Nets, insecticide, wages, equipment, transport, storage space	Adjusted financial cost plus donated insecticide	Yes	1999	US$ (and Rand)					3.82	40
	Campaign (Distrib. point not specified; assumed to be central)	CE	Thailand (Thai-Myanmar border)	Provider	Wages, material, capital cost		Yes	1994	US$					1.30^F^	41
	Antenatal Clinics	C	Kenya	Provider	Net and insecticide, transport		No	2001	US$			3.81^F ^5.26^F, L^			19

Public/Public	Campaign (Door-to-door)	C	Columbia	Provider	Wages, per diems, insecticide, transport		Yes	2001	US$		5.10^F ^(near) 12.40^F ^(far)				42

	Campaign (Fixed-site)	C	Tropical Africa	Provider	Nets, wages, allowances, transport		Yes	2003	US$	2.40^F^					22
	Campaign (Fixed-site)	CE	Kenya	Society	Insecticide, nets, wages, supplies, transport, buildings, equipment, furniture, water, time of users	Adjusted financial costs plus value of community labour	Yes	1996	US$			1.90^NC ^2.20^N^		1.4^NC ^1.6^N^	4
	Measles vaccination sites	C	Ghana	Provider	ITNs, training and supervision, transportation, community education		No	2002	US$			3.74^F^			43

Public/Mixed	Campaign – fixed site (Field trial using PHC workers & villagers)	CE	The Gambia	Society	Insecticide, wages, transport, equipment, treatment, funeral expenses	Adjusted financial costs plus time of volunteers, carers, hours lost due to mourning	Yes	1990	US$		5.65				44
	Local clinics Mobile teams CHW	C	Afghanistan	Provider	Nets, insecticide, monitoring, supervision, training, clinic overheads, wages, transport	Cost per net was adjusted (but not of other capital items)	Yes	1995	US$	2.01 1.89	1.08 0.49 1.87				45
	Campaign (Door-to-door)	CE	The Gambia (National Programme)	Society (Provider + Community)	Insecticide, wages, supplies, services, transport, equipment, awareness campaign, community capital costs incl. nets	Adjusted financial costs plus community time and water	Yes	1991 – 92	US$ (and Dalasis)		1.00^N^	3.30^N^			26
	Campaign (Fixed site)	C	Vietnam (Hoa Binh Province)	Provider	Insecticide, equipment, labour, nets and transport (purchased by community)	Adjusted financial costs	Yes	1996	US$	0.58 – 0.61^P^	0.32^P^			0.90 – 0.93	31

Mixed/Mixed	Public and private sales agents	C	Tanzania	Project costs and users' contributions			No	1998 – 99	US$	6.14 – 6.87^F^	1.72 – 2.11^F^				46
	Public and private sales agents	CE	Tanzania	Project implementation costs, user contributions, travel costs	Adjusted financial costs plus time of users, in-kind community contributions, donated inputs	Yes	2000	US$ (and Shillings)				8.30^F^	13.38		5
	General health facilities & commercial outlets	CE	Malawi	Provider	Capital and recurrent costs	Adjusted capital costs (assumed life-span as in Hanson *et al*. 2003)	Yes	1998 – 2003	US$			2.63	4.41		27

Private/Mixed	Community (Community groups)	C, CE	Kenya (Western highlands)	Provider & Society	Wages, nets, insecticide, cost-recovery included and excluded in analysis	Adjusted financial costs plus opportunity cost of MoH Government, community, and of using NGO truck	Yes	2000	US$ (and Shillings)			4.68 30.00^O^			47,48
Private/Private	Community	C	Benin (Savalou region)	Provider	Local mosquito net production, insecticide, transportation, wages		No	1993	US$ (and CFA)			10.50^F^(sales price incl. profit)			49
	Community (Community groups)	C	Kenya	Provider	Nets, netting material, insecticide, project running cost, monitoring, awareness campaign, training, transport	Some financial costs adjusted (but nets not included as capital items)	No	2002	US$			15.80^F ^14.40			9
	Formal sector retail outlet	C	The Gambia	Provider	Net (locally made), insecticide	Cost of net annualised but not discounted	No	1987	US$	1.75	0.30	2.05			50

* Delivery Point/Financing of net & insecticide (Public = Free, Mixed = Partially Subsidised, Private = Full Cost and includes payment by households)

** C = Study providing cost estimate; CE = Study carried out to establish cost-effectiveness

*** Guidelines include reference by the authors to relevant documents for guidance on costing

**** Economic cost is provided unless otherwise indicated (economic costs are derived from financial costs by excluding VAT, annualising capital costs and annualising programme development costs over expected useful life of net)

^C ^Community effect included; also called 'mass effect', see reference 51 and 52 for examples

^F ^Financial cost, rather than economic cost

^I ^Incremental cost over two year study period

^L ^Cost including leakage to non-target group

^N ^Net cost, i.e. total implementation cost minus resources saved

^O ^Cost estimate including overheads to support the NGO facilitating the ITN programme

^P ^Average cost per person protected, not per net; generally nets were single or double size

^1 ^see reference 23 for price in US$

### Technical characteristics

#### Viewpoint taken

A study's viewpoint determines the costs to be included in the analysis. Though the perspective was sometimes not explicitly stated, it was possible to infer it for all the papers after consideration of the costs included. The majority of studies were conducted from the perspective of the service provider (Table 1). The use of a societal viewpoint was limited to five of the cost-effectiveness and one of the cost studies. Less than half of the cost-effectiveness studies included the societal viewpoint among the alternatives investigated. One study examined only the patients' viewpoint.

### Types and clarity of cost measures

Financial costs of the service provider were included in all but one study, which was costed from the patients' viewpoint. Considerable variation was observed between studies in the items identified and measured. No general template could be identified by which authors had decided which inputs to include. Few studies detailed all the inputs, their unit cost, quantities consumed and reasons for inclusion/exclusion in the analysis. This observed lack of consistency and often detail could not be attributed to authors not consulting the relevant health economics literature. More than half of the studies referred to papers on costing or to one of the leading texts in this field. Most frequently quoted were Phillips *et al *[15], Creese and Parker [16], the first and second editions of Drummond *et al*. [17] and Gold *et al*. [18].

Insufficient attention was given to the cost of reaching specific target groups; in the case of ITNs these are generally children below the age of five or pregnant women. Two papers explicitly recognized this cost. Guyatt *et al*. [19] calculated two costs, one for delivery of nets to all users and a higher one for delivery to pregnant women (i.e. accounting for the proportion of nets that had been taken-up by other users). An intervention trial in northern Ghana considered it necessary to distribute nets to all family members to ensure that children were protected and considered this in the cost estimate [20].

Studies varied greatly in the way in which financial costs were adjusted to obtain economic costs (i.e. annualized and discounted) and which other economic costs, such as donated items and time of volunteers, were included. Eight studies only included financial costs. Some studies quoted the cost net of any cost savings, and one study included the community effect, i.e. the protection gained by people without a mosquito net when sleeping near ITN users. Comparison of cost estimates between studies that had used the same output was therefore not always meaningful.

### Sources of cost data

A number of studies were undertaken alongside the early efficacy trials for ITNs, which will have facilitated the collection of cost data. However, the resulting cost estimates may be an overestimate of the costs of a routine intervention because efficacy trials require close monitoring and supervision to provide reliable clinical results; in such circumstances effectiveness may also be overestimated compared to routine conditions [21]. On the opposite end of the spectrum, one study collected cost data for ITN distribution alongside measles vaccination, but did not fully account for the proportional use of campaign resources. Some studies did not collect primary data on expenditure. Instead, investigators either used results of other studies to recalculate cost estimates under different assumptions or a combination of results from other studies extrapolated to a generalized scenario [see [22]].

### Issues of time

An ITN programme generally lasts for more than one year and the capital items purchased to implement it mostly have a life expectancy in excess of one year. It is therefore useful to express capital costs as an annual equivalent, which requires judgements on the average life expectancy of each type of item and choice of a discount rate. Capital inputs that were annualised included mosquito nets, vehicles and equipment. Assumptions on the life expectancy and discount rate varied considerably between studies. Mosquito nets were considered to last three to seven years, the need to re-treat them was estimated at once or twice a year and the number of people protected by each net ranged from one to more than three. Discount rates applied to capital items ranged from 3% to 10%.

### Choice of output and outcome measure

Costs were generally quoted in US$ and in some cases also in the equivalent local currency. Two studies provided results only in local currency. Overall, five intermediate output measures were encountered: i) Cost per net delivered, ii) Cost per insecticide treatment delivered, iii) Cost per ITN delivered, iv) Cost per treated net year, and v) Cost per person protected. More than half of the studies provided only one of these; the preferred one being cost per ITN delivered. Programmes that did not distribute pre-treated nets could have distinguished the costs of delivering the net and insecticide, but often failed to report these separately. Outputs were thus not easily comparable between studies and the information provided was insufficient to calculate cost for a different output measure from that provided by the authors.

Most of the cost-effectiveness studies determined their own effectiveness data and provided one or more of a selection of outcome measures, such as cost per case averted, cost per death averted or cost per disability-adjusted life year (DALY) averted. As in case of the outputs reported, the selection of outcome measures varied between studies, with most authors reporting results for only one.

### Sensitivity analysis

Cost estimates inevitably involve some assumptions and methodological controversy. To account for this, careful analysts need to identify critical assumptions and areas of uncertainty and then re-estimate the results using different assumptions to test the sensitivity of the results and conclusions to such change. Of the studies reviewed, approximately half provided some form of sensitivity analysis. Eight (i.e. 73%) of the cost-effectiveness analysis included this component, as opposed to four (29%) of the studies that provided cost estimates.

## Discussion

The present work adds to the literature on economic aspects of the use of ITNs. A previous review covered the costs and benefits provided in sixteen published and unpublished studies from 13 countries [23]. It is assumed that the "price of bednets" quoted in this earlier review refers to the financial or economic cost per net delivered, rather than the price nets were sold for in the market. Variation in the quoted cost of mosquito nets ranged from US$ 3.00 in China to US$ 72.00 in rural Cameroon, and the cost for insecticide treatment from US$ 0.10 in China to US$ 2.00 in urban Cameroon. The assumed and observed life expectancy of nets varied from one to six years [23].

More recent results are similar, though the sources of data are different. Only four of the previously reviewed studies were included in the present review, as they were published in the English scientific literature. Here the cost per net delivered ranged from US$ 0.58 for a public sector programme in northern Vietnam to US$ 6.87 for a social-marketing programme in four areas in Tanzania. Variation in the cost of insecticide treatment was more pronounced. A study from The Gambia reports cost per treatment to be US$ 0.30, whereas delivery of treatment in remote areas of Colombia was estimated at US$ 12.40. Unfortunately, these costs could not be directly compared, because costing methodology varied too widely. The broad definition of costing inevitably captured studies with methods that would not fulfil economic standards of cost analysis. Unexpectedly, however, even studies that claimed to be costing or cost-effectiveness studies varied widely in quality.

A typology for ITN distribution and payment mechanisms recently updated by J. Webster (pers. com.) was used to categorize the studies included in this review. This indicated that the largest number of cost estimates came from programmes that use public distribution mechanisms and provide nets free of charge. Correspondingly, this category also held the largest variation in quality of the costing presented, ranging from very detailed estimates calculated as a component of cost-effectiveness studies [e.g. [4]] to "back of the envelope" figures used for advocacy [e.g. [22]]. Costing of delivery strategies categorized as mixed or private has not received much attention to date. Further cost analyses and economic-evaluations of ITN programmes are thus necessary to close this gap and to provide much needed evidence as to which delivery and payment methods, and combinations, present good value for money and should be supported in order to achieve targets outlined in the Roll Back Malaria Abuja targets [26] and the Millennium Development Goals (http://www.developmentgoals.org).

The present review also highlights the narrow perspective frequently taken to arrive at cost estimates. Authors predominantly chose the provider perspective, failing to consider the costs attributed to ITN users and society. This lack of consideration is by no means limited to the costing of ITN programmes, but has been reported for economic evaluations of interventions to control communicable and parasitic diseases [13,14]. However, user costs vary depending on the degree to which strategies shift costs from the provider to the user. For example, distribution from a central location may be relatively cheap for the programme but shifts costs to the users who need to travel to acquire ITNs. Community-based distribution systems will result in a different distribution of costs between the provider and the user. With ITN programmes generally aiming to reach certain target groups, such as the rural poor (which tend to be at highest risk of deleterious effects of malaria [e.g. [24,25]]), shifting of costs to the users should not be overlooked.

A narrow perspective also pays insufficient attention to the wastage of resources, such as leakage of nets and insecticide to non-target groups. If nets or insecticide are taken up by non-target groups, but still put to their intended use, this could be considered as adequate use of the programmes resources, because it increases overall coverage. However, if nets are used for other purposes, such as fishing or bridal wear, and if insecticide is diverted to agricultural use, then this should certainly be reflected in the ITN delivery cost. Ideally, authors should document such events, provide details on the type and scale of leakage, and calculate cost estimates including and excluding it [e.g. [19]].

This type of transparency should in fact be applied to all aspects of the cost analysis, allowing the reader to judge the reliability of the final cost estimate and, ideally, to recalculate costs under different scenarios. Transparency starts from a clear statement of the perspective taken and should be followed through by citing the source of cost data, listing the costs identified, the amounts required and their value separately, rather than as composites and by making assumptions explicit. By not adequately describing their methods of cost analysis, authors evoke the suspicion of omitting certain costs to advocate for a particular intervention [13].

Only few costing studies on ITN interventions have investigated the potential cost implications of scaling-up. Those studies that have investigated this issue have arrived at different conclusions. For example, Aikins *et al*. [26] noted a considerable difference in cost-effectiveness estimate for two types of implementation in the Gambia. When compared to a controlled intervention study, the National Impregnated Bednet Programme had difficulties controlling its field activities, which almost tripled the cost per death averted (US$ 220 *versus *US$ 620). A recent cost-effectiveness study on a nationwide ITN programme in Malawi showed that as the programme expanded and increased ITN sales over a 5-year period, costs per ITN distributed and per treated net year decreased from US$ 5.04 to US$ 1.92 and from US$ 7.69 to US$ 3.44, respectively [27]. As highlighted by the authors, further economic evaluations of other large-scale programmes using different ITN delivery methods will be required to assess under which conditions increasing returns to scale can be expected in other contexts. In any case, extrapolation from small-scale trials to the potential cost of an ITN intervention at national or international scale [22] is unlikely to be reliable, due to inevitable changes in returns to scale associated with scaling-up [28]. Authors that wish to explore the potential changes in cost when scaling-up an ITN intervention should consider the use of non-linear cost functions and other methods promoted by WHO-CHOICE [29,30].

Costing of health interventions presents a challenge in the selection of appropriate methods, but it is not too technical a task for non-economists. What investigators require is a critical attitude towards their own work and an aspiration to produce results that are meaningful in a specific context and transferable to others. The following recommendations are meant to help address some of the methodological challenges specific to ITN distribution and to make the process more accessible. Following the suggestion of Gold *et al*. [18] a reference case scenario is provided, which aims at generating comparable results from future studies.

## Recommendations

### Viewpoint

Cost analysis should be undertaken from the broadest perspective, that of society, to incorporate all costs regardless of whom incurs them [18]. Other perspectives, such as that of the provider, patient or Ministry of Health, can be included alongside this perspective.

### Costing

General costing methods are outlined elsewhere and should be consulted prior to the study. To aid generalisability and to ensure transparency, the required resources, their quantities and their value should be reported separately. Specifics related to ITN programmes are as follows.

### Resource identification

The costs of any activities undertaken to educate people to facilitate behavioural change should be accounted for, including any advertising that is part of the intervention. It should also be made clear whether an intervention provides mosquito nets or treatment only, or whether it delivers both components. For the latter, separate costs should be provided.

In areas where LLINs are being introduced alongside conventional nets the resources associated with this change, such as additional educational campaigns/materials and the amount of insecticide wasted by re-treating LLINs during net treatment campaigns should be included. For example, the malaria control programme in Uganda treats all nets while LLINs are being phased in. In 2004, an estimated 25% of all nets were LLINs, amounting to approximately 500,000 nets (A. Kilian, pers. com). In a recent campaign in 20 districts the treatment of LLINs brought for re-treatment resulted in a cost of approximately US$ 100,000 (cost includes insecticide, equipment and implementation).

For intervention trials, sensitivity analysis should consider the resources that may be required under routine programme conditions. Research costs should not be included in the intervention costs. However, it should be investigated whether the programme would operate as well if research were not carried out alongside it. If research staff are considered to be essential for the intervention, then this cost should be included [e.g. [5]]. Another parameter that should be subjected to sensitivity analysis is the proportion of shared resources in cases where distribution is carried out as a component of another programme/campaign. Here it is particularly important to be explicit about the criterion used to attribute resource use (e.g. % time, % value of resources), as the basis for the breakdown is always arguable. For collaboration between organisations (e.g. local NGO & international NGO), resources including overheads that are relevant to the programme need to be identified. The final cost estimate needs to reflect the fact that, for example, a programme was made feasible because an international NGO facilitated procurement, management, fund raising, etc.

Programme management resources need to be identified and clearly specified, even if the main purpose of the study is to compare two interventions provided by the same programme (e.g. ITN/IRS). To ensure generalisability it needs to be clear what capacity is assumed to exist and whether authors have calculated average costs for delivery of each intervention or the incremental cost of adding an intervention to an existing programme (e.g. ITN delivery alongside vaccination campaigns). In areas where a mix of locally made (cotton) and imported (polyester, polyethylene) nets is being impregnated, the extra resources required to treat the former need to be considered, as these nets take up at least twice the amount of insecticide [e.g. [31]].

### Resource measurement

An attempt should be made to measure and report the leakage of resources, to be able to differentiate between measured outputs (e.g. nets delivered) and outputs that reach the intended target. To inform policy, it is essential to determine the degree to which different approaches to targeting achieve their aim and at what cost.

It should be established how long nets last under local conditions. Observations to date indicate that assumed life expectancies of more than three years are unrealistic (except in the case of polyethylene nets such as Olyset^®^). In general, a relevant timeframe needs to be used for the costing. As most projects last for more than one year there may be issues around price level adjustments, so that all the costs can be expressed in the same year, and because of lumped expenditure (i.e. high start-up costs, but falling over time). The general recommendation with regards to tracking of costs is to select a follow-up period that does not bias the analysis in favour of one intervention over another [17].

### Resource valuation

Mosquito nets need to be treated as a capital item. Annualization based on a 3 years life span is recommended. Should surveys indicate otherwise, costs for observed and assumed 3 year duration should be provided. To all capital items a discount rate of 3% should be applied, to be consistent with the rate used by the World Bank [32]. Sensitivity analysis should also include 0%, 5% and 10%, to presents costs in their undiscounted form, as well as at higher rates chosen by some analysts.

When comparing alternatives, for example ITNs and IRS, the same or equivalent components for their delivery need to be costed. When using cost estimates reported in other studies, these need to have been presented transparently so that they can be adapted (e.g. by applying local prices to standard quantities). Otherwise, costs for a specific study need to be identified, measured and valued and to be assessed for consistency with other authors' estimates. For programmes that recover costs through user contributions, double counting needs to be avoided. For the societal perspective these costs should be included, whereas if the provider perspective is taken they should be excluded [e.g. [5]].

### Data analysis

To reflect the uncertainty in measurements, a sensitivity analysis needs to be carried out on (at a minimum): discount rate, frequency of net impregnation, procurement cost of insecticide & net, number of people protected per net, life span of net. Where it is necessary to estimate a share of resources contributed from other programmes or interventions, the assumptions used should be subjected to sensitivity analysis.

### Reporting of results

Cost estimates should be provided in US$ and local currency, and the year to which costs were adjusted needs to be specified. Projects producing nets locally for sale need to report on the cost of making/treating nets, not just the price at which these were sold to users, which may not fully measure the opportunity cost.

The cost per ITN delivered should be reported, as cost per person sleeping under a net varies depending on cultural practices and as cost per case of malaria prevented depends on the incidence of malaria. Both are useful for policy decisions in a given context, but the cost per net delivered is best suited for comparing delivery strategies. Cost-effectiveness studies should not just provide net costs (i.e. subtracting resources saved from total implementation costs), as these cannot be compared to results from costing studies.

### Discussion of results

To put cost-estimates for a particular method, or combination of methods, into perspective, it is recommended that they are compared to the per capita expenditure on government health services in the country/region. This does not improve on the comparability of costing studies between countries, but will help readers to assess the affordability of the intervention [e.g. [3]]. When reporting results, one should beware of the assumptions required to extrapolate these to calculate costs of scaling-up an intervention. To date, studies that have investigated the cost of scaling-up health interventions are limited. Available data indicate that scaling-up costs are highly specific to both the type of intervention and its particular setting [30]. Without further study of potential additional cost and without considering factors such as human resources, geography and infrastructure, extrapolations are not valid because they assume constant marginal costs.

### The recommendations are summarized in Table 2

**Table 2:** Recommendations for calculating and presenting cost results

Viewpoint	Use societal perspective. Sub-analyses can focus on specific perspectives such as provider, patient or Ministry of Health
Output	Clarify whether intervention delivers nets or treatment or both Calculate net and treatment costs separately
Resource identification	Include all costs of behaviour change activities (including advertising) Include any costs of treating LLINs in campaigns Exclude research costs Include relevant overheads of collaborating organizations (e.g. NGO contributions to procurement, management, etc) Clarify what management capacity is assumed to exist and whether calculating average cost or incremental cost of adding intervention to an existing programme
Resource measurement	Attempt to measure and report leakage of resources (e.g. nets used by individuals outside target groups) Establish average lifespan of net under local conditions
Resource valuation	Treat nets as a capital item. Base case should assume life expectancy of 3 years and use discount rate of 3% Avoid double counting of user contributions where cost recovery applied, but ensure these are counted as user contributions when disaggregating costs by source
Sensitivity analysis	Conduct sensitivity analysis on: discount rate (0%, 5%, 10% at a minimum); frequency of net impregnation, procurement cost of net and insecticide; number of people protected per net; lifespan of net Consider impact of research where this is conducted alongside a programme on programme effectiveness (see ref 5) Vary proportion of shared resources where distribution carried out as part of another programme/campaign
Reporting of results	Provide costs in US$ and local currency Specify year in which costs calculated/adjusted Report cost per ITN delivered and cost per person sleeping under a net
Discussion of results	Compare costs with per capita government health expenditure to aid assessment of affordability Be cautious in using cost estimates to scale up, and make explicit assumptions about whether marginal cost constant, increasing or decreasing

### The reference case

To provide comparability between ITN costing studies it is suggested that analysts include a reference case scenario. If future costing studies adhere to this practice, it will finally be feasible to compare the costs of different options for ITN delivery. At the same time, authors maintain the freedom of analysing their data from other viewpoints that may be more relevant to their specific context. Based on the above recommendation the use of the following reference case scenario is proposed (Table 3).

**Table 3:** Reference case scenario

**Parameter**	**Suggested Reference Scenario**	**Explanation**
Perspective	Societal	To include all costs, not just those of the provider or patient.
Currency	US$	A cost estimate in US$, indicating the year of conversion should be provided in addition to local currency
Life-span of mosquito net	3 years	Assumptions have varied from 3 to 7 years, but field observations increasingly indicate a relatively short life span of polyester nets. Olyset^® ^nets (made of polyethylene) are more durable and should be considered separately.
Re-treatment	Annually	Treatment of mosquito nets with modern pyrethroids is generally assumed to last 6 – 12 months. Evidence for LLINs (Olyset^® ^and PermaNet^®^) indicates that treatment lasts for the life span of the net [7, 8]. While LLINs are phased in, they may be retreated alongside conventional nets. It should be investigated if this is the case.
Cost data	Include: All intervention costs (e.g. mosquito nets, insecticide, wages, transport, advertising, etc.) All time cost, including care giving (formal/informal) and volunteers Transportation and other non-medical services Administrative costs for sick leave and for other transfers Donated items	Costs for these and possible other ingredients (depending on programme specifics) should be collected. Costs can only be excluded once it has been established that they are insignificant in the context of the analysis [see reference 17 and 18 for further guidance). Quantities and prices need to be presented separately
Revenue	Value and include	From the societal perspective, funds from cost-recovery need to be included. This needs careful attention to avoid double counting. Clearly indicate cost-recovery when presenting results.

Adjustment of financial costs to calculated economic costs		

Annualisation	Life expectancy of capital items as specified above	To obtain an equivalent annual cost for each capital outlay, an annuitization procedure needs to be followed. This requires an estimate of the life expectancy of each capital item and a decision on the discount rate to be used (see below).
Discount rate	3%	Base-case calculations should use 3%, to be consistent with World Bank recommendations [32]. This should be varied in the sensitivity analysis, e.g. from 0 – 10%.

Reporting of results		

Cost estimate	Cost per net ITN delivered	For programmes delivering untreated nets and insecticide treatment, the cost of both components should be quoted separately. Indicate whether the cost per ITN is the composite of the two costs or is achieved at lower/higher cost

## Conclusion

The cost of different ITN delivery strategies is important when deciding which ones to scale-up, yet it is one of the knowledge gaps remaining to be filled [10]. Many strategies have not been costed at all and only some of the existing cost-estimates have been derived using appropriate methods. Well-conducted studies have often used outputs that are difficult to compare. A template for costing of ITN interventions and reporting on results does not exist.

The limited role of health economics in generating essential evidence for scaling-up of ITNs cannot be explained by the absence of general guidelines or other supporting literature. More likely it partly results from the lack of ITN specific recommendations, which has led to the observed variation in methods and outputs, and partly from some authors being reluctant to use any economic methods, because these are misconceived as not applicable to health care [33]. Until these obstacles are overcome, decision makers will lack important data to guide their efforts of scaling-up coverage.

To advocate for the use of improved and standardised methods, this review has drawn attention to the variations in approaches taken to costing ITN programmes, and consequently of study outputs, and the limitations of these. This will hopefully allow ITN programme staff and their donors to recognise the potential impact that costing of their work could have, if results were readily comparable to other studies and could be interpreted in other contexts. The suggested Reference Case scenario is meant to further assist standardisation of new costing initiatives.

## Authors' contributions

Kara Hanson suggested the topic for the review, provided advice on structure and content and was involved in re-drafting the paper. Jan Kolaczinski reviewed and summarised the literature, and wrote the first draft of the paper.
